# Electromagnetic Wave Absorption in the Human Head: A Virtual Sensor Based on a Deep-Learning Model

**DOI:** 10.3390/s23063131

**Published:** 2023-03-15

**Authors:** Paolo Di Barba, Łukasz Januszkiewicz, Jarosław Kawecki, Maria Evelina Mognaschi

**Affiliations:** 1 Department of Electrical, Computer and Biomedical Engineering, University of Pavia, Via Ferrata 5, 27100 Pavia, Italy; 2 Institute of Electronics, Lodz University of Technology, Al. Politechniki 10, 93-590 Lodz, Poland

**Keywords:** convolutional neural network, bioelectromagnetic analysis, surrogate model, FDTD simulations

## Abstract

Determining the amount of electromagnetic wave energy absorbed by the human body is an important issue in the analysis of wireless systems. Typically, numerical methods based on Maxwell’s equations and numerical models of the body are used for this purpose. This approach is time-consuming, especially in the case of high frequencies, for which a fine discretization of the model should be used. In this paper, the surrogate model of electromagnetic wave absorption in human body, utilizing Deep-Learning, is proposed. In particular, a family of data from finite-difference time-domain analyses makes it possible to train a Convolutional Neural Network (CNN), in view of recovering the average and maximum power density in the cross-section region of the human head at the frequency of 3.5 GHz. The developed method allows for quick determination of the average and maximum power density for the area of the entire head and eyeball areas. The results obtained in this way are similar to those obtained by the method based on Maxwell’s equations.

## 1. Introduction

In recent years, surrogate models based on deep neural networks are becoming popular in computational electromagnetics [1,2]. In fact, the development of digital computers has allowed problems that were previously unsolvable to become tractable, leading to the development of so-called digital twins of the physical system in the virtual environment. In fact, the basis of current machine learning systems is the representation of the mapping between parameter and performance spaces by means of a large number of examples to generate a response surface. One method of achieving this goal is based on neural network architecture, which has been recently applied to problems in the area of electromagnetic analysis and synthesis with some success [3]. In particular, convolutional neural networks make it possible to process a picture, e.g., a field map in a region, as the input [4]. A fundamental requirement of such an approach is the existence of a large and suitable database of examples; while still expensive to achieve, this is now feasible and opens up the possibility of using deep-learning systems to generate a black box model of practically any relationship, including the ones ruled by electromagnetic equations. In this paper, we propose a convolutional neural network (CNN) for modeling the power density map in a very complex domain as the human body is. Since full-wave simulations are computationally expensive [5], the proposed method could be a promising solution to this problem.

**Figure 1:**
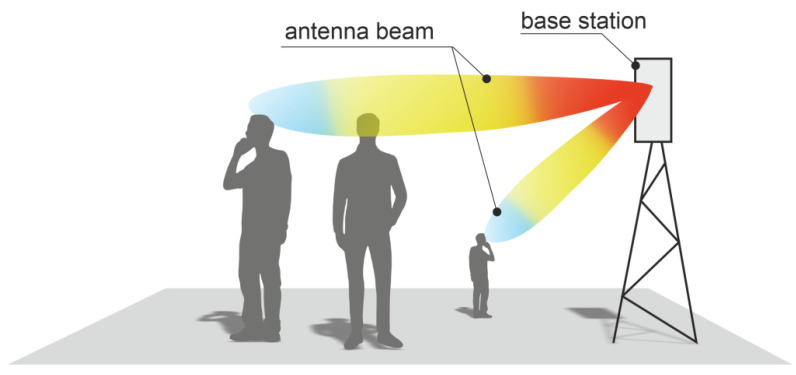
Problem scenario: exposure to 5G base station with multiple beams.

The simulation of electromagnetic wave energy absorption by the human body is a key issue, especially when considering 5G base stations which utilize an antenna array with dynamically formed and narrow beams, which change their orientation in space and time. In this technology, the base station antenna can emit multiple beams directed at different users, as shown in Figure 1. Unlike wireless systems of previous generations, the radiation pattern of such antennas changes dynamically. Therefore, the parameters of body exposure to electromagnetic waves also change, both due to the energy density of the beam and the direction from which the wave comes. This makes it necessary to consider many scenarios when analyzing the body’s exposure to radiation, taking into account a wide range of angles from which the wave energy arrives [6]. Analyses should be carried out for different frequencies used by 5G systems. In this article, we adopted the 3.5 GHz frequency for further analysis, as it is currently used in many European countries.

In our research, we use power density as the measure of the intensity of an EMF since it is often used to assess exposure to radiofrequency (RF) fields, such as those from mobile phones and base stations. Power density is a useful measure when the EMF source is at a distance from the body, and the EMF is relatively uniform over a large area, such as in the case of outdoor RF sources. In such cases, the exposure can be estimated by measuring the power density at a certain distance from the source. This measure is used in the regulations concerning human body exposure. The International Commission on Non-Ionizing Radiation Protection (ICNIRP) provides guidelines for limiting exposure to EMFs, including power density limits for different frequencies and types of exposure. In [7] a comprehensive review of the scientific literature on the health effects of electromagnetic fields provides recommendations for protecting against both short-term and long-term effects of exposure to electromagnetic fields. Directive of the European Parliament and of the Council [8] deals with the minimum health and safety requirements regarding the exposure of workers to the risks arising from physical agents (electromagnetic fields). The directive sets out requirements for assessing and controlling exposure to electromagnetic fields in the workplace, and it uses a variety of measures to do so, including power density.

In the paper, we propose a virtual sensor that is able to model the power density in a cross-section of the human head at eye-level. The word “virtual” in this sense means the model that utilized a convolutional neural network instead of full-wave computer simulations based on Maxwell equations for electromagnetic analysis. Instead of estimating the power density distribution in the body, this sensor is capable of estimating the maximum and average power density level for the head and the eye regions. This is justified by the fact that these parameters are crucial for assessing the safety of people exposed to electromagnetic waves. Even if the network was trained with the results obtained with finite-difference time-domain (FDTD) numerical method, after that it can autonomously estimate the parameters that describe power density level. It can perform a very rapid estimation for any direction of incident wave that is substantially faster than classical approach with FDTD.

In the introductory section of the manuscript, a brief introduction to the issue is given. Special emphasis is placed on the working principle of 5G technology. The impact of this new 5G technology on the electromagnetic exposure is briefly and simply explained. The novelties of the manuscript in this section are clearly indicated. In Section 2 entitled “Materials and Methods”, the electromagnetic problem is adequately explained. Furthermore, gathering datasets were considered. In this part, different regions of the head at different angles of the incident electromagnetic wave were considered. Finally, the architecture of the used convolutional neural networks (CNN) is explained. The results are presented in Section 3 of the manuscript. The presented results undoubtedly indicate that the proposed approach gives satisfactory results of average and maximum power density. An adequate discussion of the results is given in Section 4. The novelties of this work are sublimated in the conclusion. Conclusions are adequately conceived based on the results. Additionally, the future research of the author is clearly indicated.

## 2. Materials and Methods

### 2.1. The Electromagnetic Problem

The considered problem includes the phenomenon of electromagnetic wave energy absorption by the human body. This is an important issue from the point of view of designers of wireless communication systems. Currently, numerical methods based on Maxwell’s equations such as finite-difference time-domain (FDTD) are used for this purpose. FDTD is capable of calculating electromagnetic fields in complex objects, and the human body is no exception. It is a recommended method for calculating the effects of EM waves on the human body [9]. To apply this method to the analysis of exposure of human to electromagnetic wave, the body has to be discretized into unit cells (voxels) with respect to the interior structure representing organs made of different tissues. The electrical properties of human tissues are available in the literature for a wide range of frequencies [10,11] and in the software that implements this method they are assigned to the discretized model of human body. In this method, due to numerical stability, the voxel size should be less than 0.1 of the shortest wavelength *λ_min_* in the considered media. As a consequence, for high-frequency simulations (for which the wavelength is small), a model of the human body has to be characterized by small-size voxels, and the need to use millions of voxels for body regions originates; indeed, the computational burden is proportional to the number of voxels used. The wavelength of electromagnetic wave in dielectric media can be calculated as follows:

(1) $$\lambda_{min}=\frac{c_{0}}{f_{c}\cdot \sqrt{\mu_{r}\cdot \varepsilon_{r}}}$$

where:

*λ_min_*—shortest wavelength in the model.
*c*_0_—velocity of light in vacuum.
*f_c_—*wave frequency.
*μ_r_—*model relative permeability.
*ε_r_—*model relative permittivity.

The shortest wavelength occurs in the region of the model with the highest permittivity. Human eyes contain high permittivity tissue, that is vitreous humor for which *ε_r_* = 67 at 3.5 GHz. At this frequency the wavelength in this region *λ_min_* is equal to 10.4 mm. The recommended voxel size should be then 1 mm.

In our research, we have utilized the FDTD method and heterogeneous model of the human head to obtain data for network training. Specifically, Remcom XFdtd version 7.9 software [12] along with a body model exhibiting 1 mm voxel size was applied. Specifically, the used model of the head consists of 25 tissues with electric properties characterized by Cole–Cole parameters [13]. This a model that was made using information from body scans obtained from the magnetic resonance technique at the Hershey Institute [14,15].

**Figure 2:**
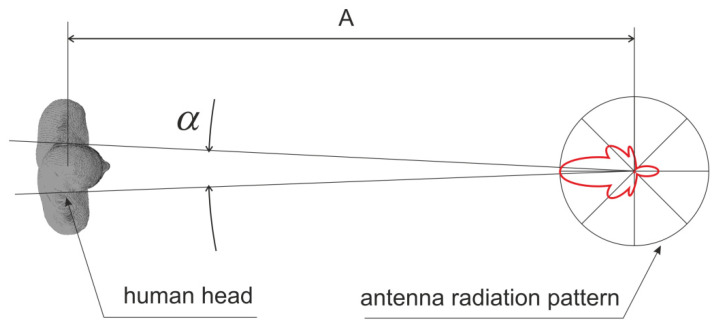
Human head exposure related to the base station antenna.

In our research, we focus on modeling the interaction of electromagnetic waves and the human body. Based on the distribution of power density in the head area obtained from the Maxwell equations-based numerical method (FDTD), we create a new model, which is a trained neural network. These studies require a lot of input data, such as FDTD simulation results. In order to carry out our research, we had to make simplifying assumptions. In further research, we do not consider all variants of body exposure to electromagnetic waves but only those that we consider important for the potential application of the model, i.e., quick verification of people exposition to radiation from base stations. The radiation from a terminal to the user head is not covered in our work because it would require the analysis of the radiation from its antenna. In this case, the head is very close to the terminal (in the so-called near field) and affects the parameters of the antenna. To model this phenomenon, we would have to use the numerical model of the terminal antenna as the wave source. Since there are some design differences between terminals and their antennas, our research would be difficult to generalize. The second reason is that the antennas of 5G terminals have dynamically controlled radiation pattern, and therefore it would be necessary to analyze many cases with different radiation characteristics of such an antenna. Our research assumption, which we adopted consciously, is the exposure of the body to radiation from the distant base station and free space propagation of the electromagnetic wave. In this case, we neglect the wave components reflected from obstacles and the ground. The source of energy is the main beam formed by the directional antenna of the base station, as presented in Figure 2. In the considered case that refers to a typical scenario of mobile telecommunication system, the human head is very far from the base station. The A parameter in Figure 2 could be equal to 50 m, in the case of a person rather close to the base station. Being the width of the head approximately 0.2 m, the α angle in Figure 2 is 0.2°. Hence, we can assume that the antenna gain is the same for the whole head. Because the antenna is far from the head, we can treat it as a point source, which produces a plane wave of constant power density for the entire angle that covers head. This is a standard approach in analysis and design of mobile communication systems.

In the considered case, the person exposed (human head) remains in the far-field region of the antenna radiation. The far-field region (simply far zone), also known as the radiation zone or Fraunhofer zone, is a region of the electromagnetic field surrounding an antenna where the radiation pattern becomes essentially independent of the distance from the antenna. In other words, the far zone is a region of space where the electric and magnetic fields of the antenna radiation appear to be plane waves, and the angular distribution of the fields does not change significantly with the distance from the antenna. The far zone of antenna radiation begins at a distance from the antenna where the electric and magnetic fields are predominantly composed of transverse waves, meaning that the fields are perpendicular to the direction of propagation. The boundary between the near zone and the far zone is typically defined as the point at which the distance from the antenna is much larger than the size of the antenna itself. Its smallest radial distance *r* is given by 2 [16]:

(2) $$r=\frac{D^{2}}{\lambda }$$

where:

*D*—the largest dimension of the antenna.
*λ—*the wavelength.

In practice, the distance at which the far-field condition is met depends on the size and geometry of the antenna and the frequency of the radiation. For typical base station antennas operating in the range of several hundred MHz to several GHz, the far-field distance is on the order of several meters to tens of meters.

The far zone is an important concept in antenna theory, as it is the region of space where the radiation pattern can be accurately measured or predicted [16]. It is also the region where the majority of the radiated power is transferred from the antenna to the surrounding environment. In studies of body exposure to base station radiation, the far-field assumption is typically justified based on the distance between the body and the base station antenna. There are many papers that assume the far zone in studies of body exposure to base station radiation, as it is a commonly used assumption in the field of electromagnetic field (EMF) exposure assessment. In [7], the guidelines for limiting exposure to RF fields from base stations are presented. In this paper different reference level application rules have been set for exposure in the far-field, radiative near-field, and reactive near-field zones. In [17], all exposure calculations are performed for far-field exposure. The paper [18] presents the investigation on specific absorption rate and temperature rise in an anatomically based human model for RF far-field exposure. Paper [19] presents a recommended practice that specifies techniques and instrumentation for the measurement and computation of potentially hazardous electric, magnetic, and electromagnetic fields both in the near-field and the far-field of the source. Among other numerical methods mentioned, FDTD calculations have been recommended there because it was extensively validated for both far-field and near-field sources. The above examples of the literature sources justify the assumptions we made regarding the use of a plane wave as an energy source in our model.

**Figure 3:**
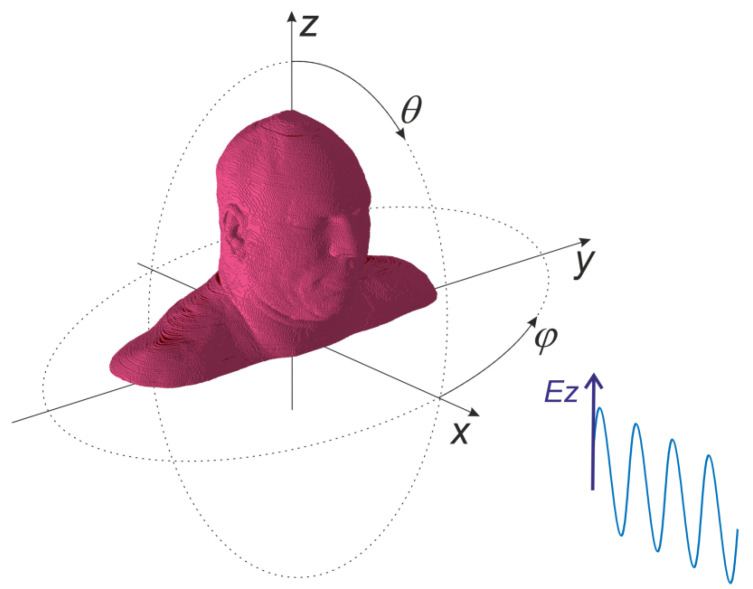
Geometry of the experiment with vertically polarized incident wave.

The field analyses were carried out in order to obtain the power density distribution due to the exposure to a vertically polarized electromagnetic wave at 3.5 GHz frequency, which is typical of 5G wireless telecommunication systems. As presented in Figure 3, the direction of wave incidence was controlled by mean of 2 angles: *θ* in vertical (z-x) and *φ* in horizontal (x-y) plane. The amplitude of incident wave was constant, and the absolute value of electric field component was equal to 10 V/m.

### 2.2. Generating Datasets

**Figure 4:**
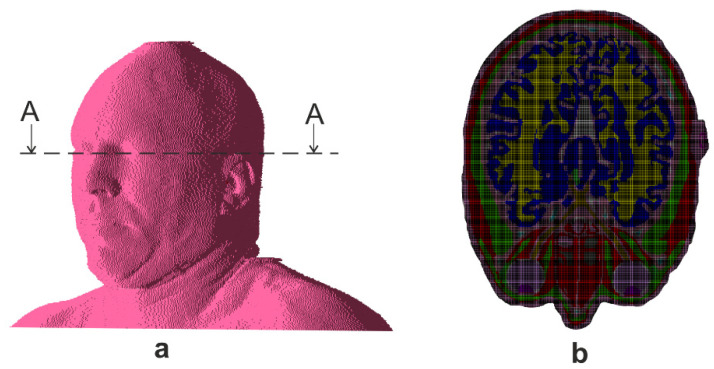
Location of Power density virtual sensor: (**a**) with reference to the head, (**b**) FDTD model cross-section in A-A plane at the sensor level.

In view of a deep-learning based model, a family of numerically evaluated power density distributions obtained for different angles were utilized. Considering the particular sensitivity of human eyes to EM waves, for which acceptable power levels are usually smaller than for the rest of the body, we gathered the data for the cross-section of the model on the level of eyeballs (Figure 4). The power density distribution was simulated for a broad set of scenarios in which wave incidence angles were set up. Specifically, a database of e.g., N = 2664 solutions was calculated by varying the values of *φ* in the range from 0° to 355° with 5° step and *θ* angle from 0° to 180° with the same step size. The range of elevation angle *θ* from 0° to 90° degrees corresponds to the location of the wave source above the user. For *θ* angles from 90° to 180° the source is below the exposed person, which may correspond to the situation of a person in a tall building. In particular, this last range of values has been used, and, all in one, N = 1387 samples (73 *φ* angles × 19 *θ* angles) have been used for the CNN training.

The FDTD based model solves the forward field problem; i.e., at a given set of incidence angles, it finds the corresponding power density distribution. The computational cost of this is relatively high, depending on the mesh density. The head region corresponds to 56,476,720 voxels with the voxel size equal to 1 mm. Accordingly, the simulation requires 2 GB of computer memory, and the simulation time is approximately 4 min on Nvidia Quadro P5000 GPU.

### 2.3. Designing Deep Neural Network Models

For solving the forward field problem via a surrogate model, the synthesis of various architectures of CNN is considered in a comparative way.

#### 2.3.1. Forward Problem

Considering a linearly polarized (vertical) electromagnetic wave at 3.5 GHz with the amplitude of electric field component equal to 10 Vm^−1^ and given the direction of the incident wave by means of *φ* and *θ* angles, find the power density map in the horizontal plane of head region crossing the eyeballs.

In particular, we note that the formulation of the Deep Neural Network (DNN) architecture gives rise to an up-sampling problem which is non-trivial to solve. In fact, we start from a vector of source parameters i.e., frequency, amplitude, and direction angles (low dimension), and we aim at recovering the power density map in the whole head cross-section (high dimension) for a given voxel size. Therefore, in order to circumvent the difficulty, the formulation of a reduced forward problem is considered.

#### 2.3.2. Reduced Forward Problem

**Figure 5:**
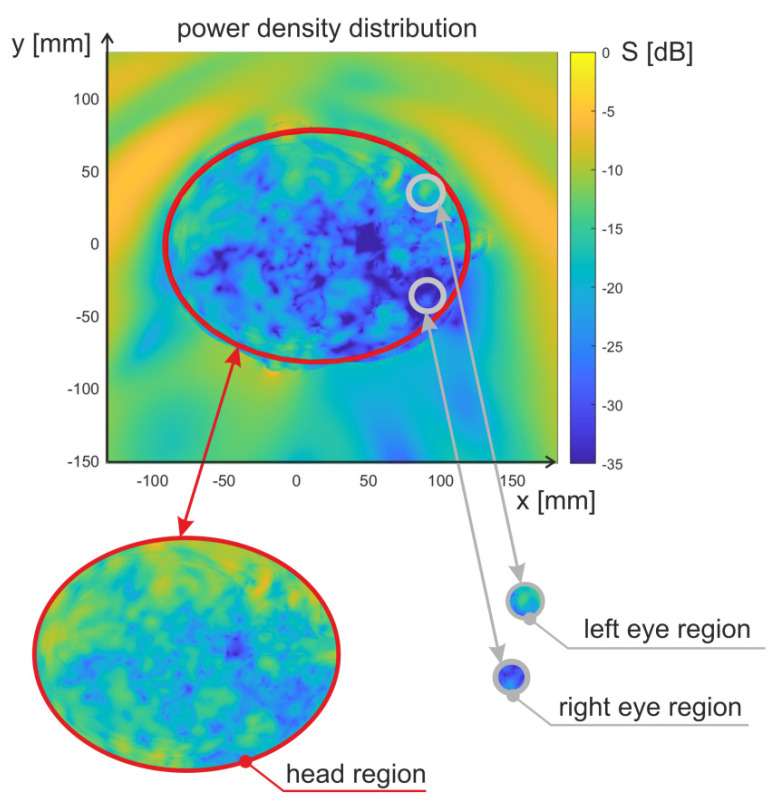
The head region and eye regions indicated in the power density distribution obtained from FDTD simulations.

Under the same assumptions as above, find average and maximum values of power density in three regions: left eye, right eye, and whole head, respectively. The head region considered for the power density calculation is the area of an ellipse inscribed in the cross-sectional area of the head. The parameters of the ellipse were selected in such a way as to cover as many body tissues as possible. The length of major axis (parallel to x axis in Figure 4) is equal to 210 mm while the length of minor axis (parallel to y axis) is 160 mm. In turn, the eye region is the area of an ellipse inscribed in the cross-sectional area of the eyeball. The parameters of the ellipse were selected in such a way as to cover the area of the eye sclera tissue. For the left eye, the length of major axis is 24 mm, and the minor axis is 23 mm. For the right eye, the major axis length is 25 mm, and the minor axis length is 22 mm. In Figure 5 the head region and eye regions are presented along with the power density distribution obtained from FDTD simulations.

**Figure 6:**
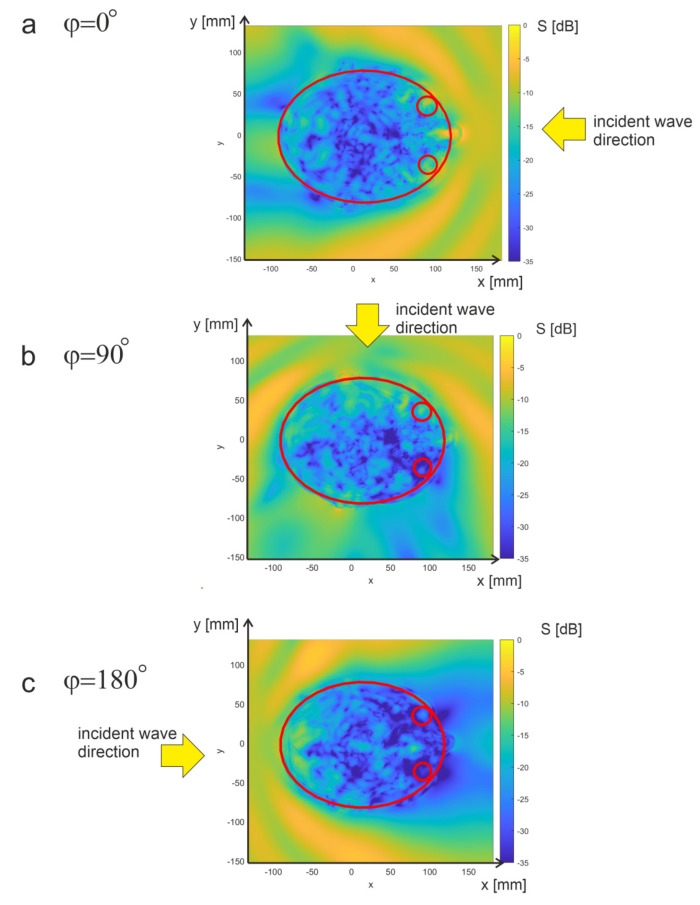
Power distributions obtained for *θ* = 90° and different angles in the horizontal plane. The head and the eyes are highlighted with red lines.

The power density distribution at different points in the head area depends to a large extent on the direction of the incident wave. Figure 6 presents exemplary results obtained for exposure of the head to a wave emitted from various directions. The results are shown for a wave propagating in the horizontal plane (x-y) for which *θ* = 90°. The simulation results are presented in the cases of a wave propagating from the front of the head, side, and back, respectively. For each of these cases, there are different sub-regions in which the maximum values of the power density occur. For an exposure with an *φ* angle of 0°, the maximum is at the front, while for an exposure with an *φ* = 90°, the maximum is on the left side of the head. In the case of exposure to the propagating wave from the side of the head vein (*φ* = 180°), there is an area with a maximum value in the posterior part.

**Figure 7:**
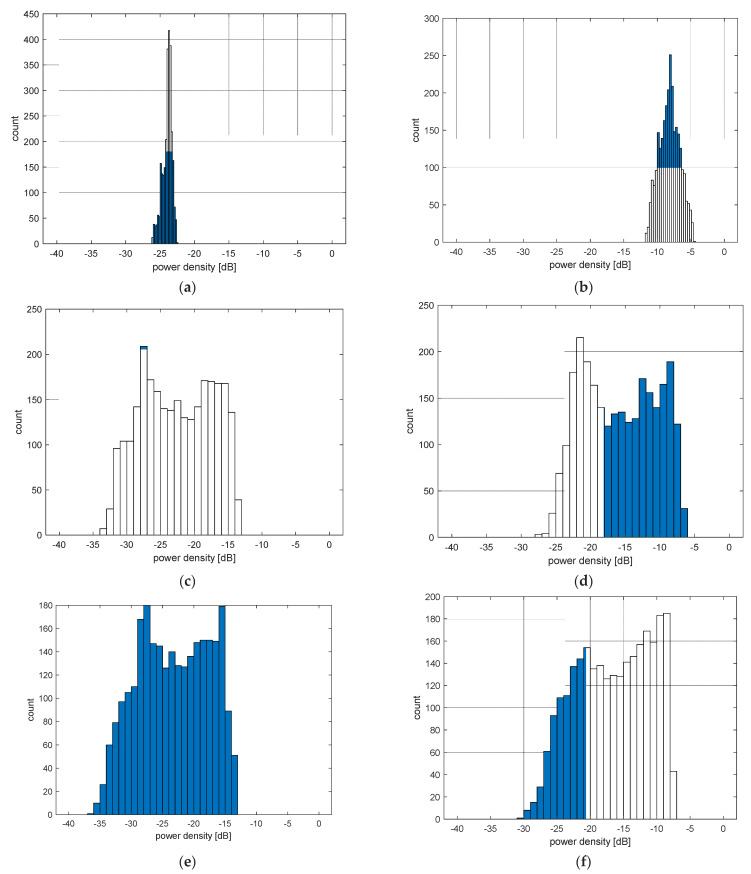
The histogram of average (**a**) and maximum (**b**) values of power density for the head region, of average (**c**) and maximum (**d**) values of power density for the left eye region and of average (**e**) and maximum (**f**) values of power density for the right eye region.

Figure 7 shows the frequency of occurrence of individual average and maximum power density values obtained for different areas using histograms. For this purpose, the simulation results for all *θ* and *φ* angles were analyzed (2664 simulations). The average power densities for the whole head area are concentrated around the value −23.7 dB, which occurred in 417 simulations. The most common result of the mean value for the left eye was −27.5 dB, but in this case the distribution is not as concentrated as it is for the whole head area. The same is true for the right eye, for which the most common value is also −27.5 dB, but scores from −30 to −15 dB have a very similar frequency of occurrence.

Similar dependencies also occur for maximum values. For the whole head area, the distribution is concentrated around the most common value of −8 dB, but compared to the distribution of average values for this area, the variance is higher. The distributions of the maximum values for the eyes are less concentrated, similar to the mean values. For the left eye, the most common value is −21.5 dB, but values −22 to −8 dB are characterized by high occurrence. For the right eye, the most common peak is −8.5 dB, and values from −25 to −8 dB are common in the results.

Specifically, the regions of eyes and head were approximated by means of an elliptical contour: in each elliptical region Average Values (AV) and Maximum Values (MV) of induced power density were calculated for the purpose of training a CNN. This way, a substantial data compression was obtained, namely:

- Input data: (2,1) vector [*φ θ*]^t^ incident wave direction and electric field value
- Output data: (3,2) matrix

[AV_left eye_       MV_left eye_

AV_right eye_      MV_right eye_

AV_whole head_  MV_whole head_]

#### 2.3.3. CNN-Architecture

**Figure 8:**
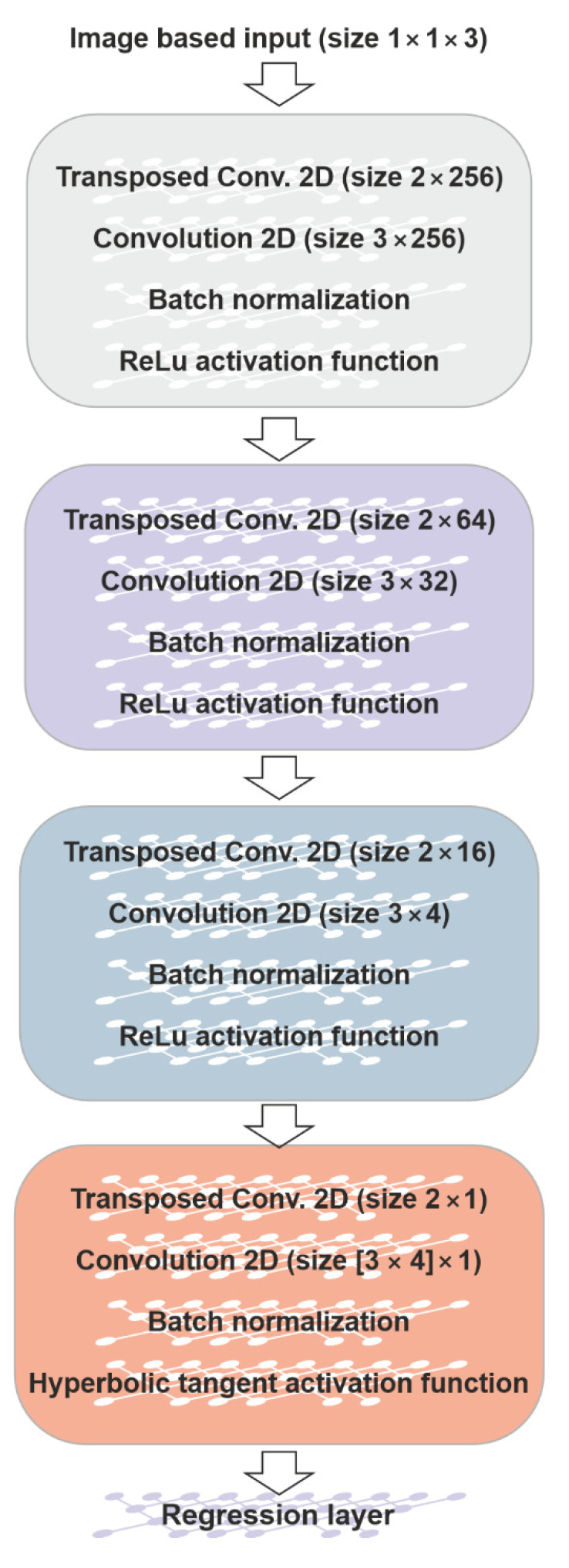
The architecture of the CNN trained for solving the reduced forward problem.

For solving the forward problem, a CNN trained from scratch is used. The CNN is composed of 18 layers, in which a block made of 4 layers and repeated 4 times can be recognized. The block is composed of a transposed convolutional layer, a convolutional layer, a batch normalization layer [20], and an activation function, as shown in Figure 8. In the first 3 blocks the ReLU function is used: it is one of the most used activation functions for CNN because it showed good performance in training this kind of neural networks in terms of preventing overfitting [21]. In turn, the last block is characterized by a hyperbolic tangent (tanh) activation function which gives a value in the range [–1, 1] as the output. In spite the tanh activation function is computationally more expensive than the ReLU activation function, it allows to obtain strong gradients during the training and therefore a fast update of the CNN weights. Moreover, the output of tanh is symmetric around zero, helping the convergence speed.

Moreover, the transposed convolutional layers are characterized by filters of size 2 × 2. The number of filters vary from 256 to 1. The convolutional layers are characterized by filters of size 3 × 3 (the first three blocks) and 3 × 4, the last one. The number of filters varies from 256 to 1, depending on the block. The convolutional layers have stride equal to 1 and padding set as “same”, which means that the dimensions of the input and the output of the layer are the same. In turn, the transposed convolutional layer allows the enlargement of the dimension of the image because the output of the layer is larger than the input. Hence, from the beginning to the end of the CNN, the image is enlarged, and the filters decrease from 256 to 1.

Eventually, in order to solve the regression problem, at the end of the network a regression layer is used. The architecture of the CNN so implemented allows to obtain a 3 × 2 matrix as output.

In order to compare the performance of the CNN with a classical approach, a Fully Connected Neural Network (FC-NN) has been implemented and trained. The FC-NN architecture is shown in Table 1.

This FC-NN has the same number of layers of the proposed CNN and the same number of weights to identify (about 680,000 weights).

In order to evaluate the accuracy of the CNN training, different metrics are used. The absolute value *D* of the discrepancy between true and predicted values is

(3) $$D_{i}=\left|Y_{i}-{\hat{Y}}_{i}\right|$$

with $Y_{i}$ the true value and ${\hat{Y}}_{i}$ the predicted value. The average value MEAN is then

(4) $$\mathrm{MEAN}=\frac{1}{n_{v}}\sum_{i=1}^{n_{v}}D_{i}$$

with *n_v_* the number of samples in the validation set.

Moreover, the standard deviation (SD)

(5) $$\mathrm{SD}=\sqrt{\frac{1}{n_{v}}\sum_{i=1}^{n_{v}}{\left(D_{i}-\mathrm{MEAN}\right)}^{2}}$$

is calculated.

Then the Root Mean Square Error (RMSE)

(6) $$\mathrm{RMSE}=\sqrt{\frac{1}{n_{v}}\sum_{i=1}^{n_{v}}{\left(Y_{i}-{\hat{Y}}_{i}\right)}^{2}}$$

is also used.

## 3. Results

For the CNN training, a database of 1387 samples is used, and the 80/20 rule, i.e., 80% of the dataset for the training set and 20% for the validation set, is applied. The ADAM solver, i.e., an optimization algorithm based on the stochastic gradient descent, is used, and 800 epochs, a batch size of 32, and an initial learning rate of 10^−6^ are considered for the training.

**Figure 9:**
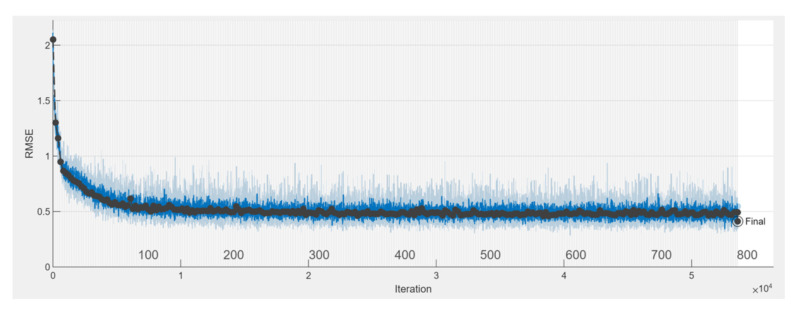
Trend of RMSE during the training (blue—training set, black—validation set).

The Root Mean Square Error (RMSE), i.e., the standard deviation of the residuals (prediction errors) versus the iterations, is shown in Figure 9.

Residuals are a measure of how far from the regression line data points are; RMSE is a measure of how spread these residuals are.

**Figure 10:**
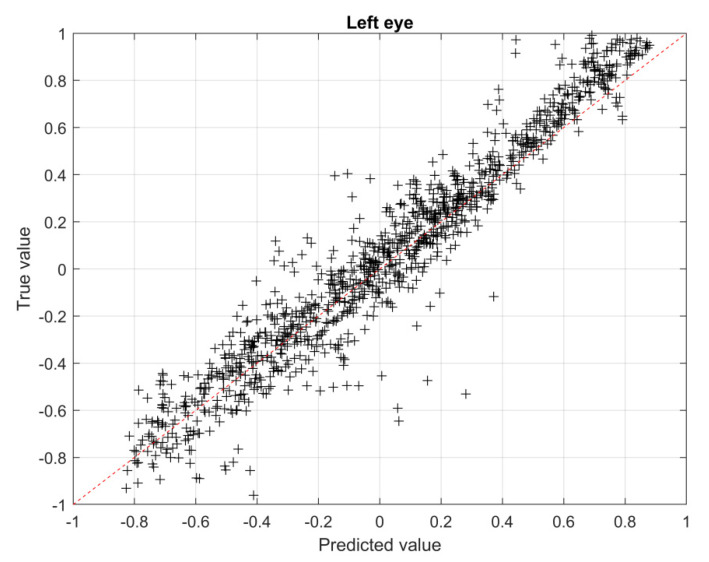
True vs. predicted values for the left eye region.

**Figure 11:**
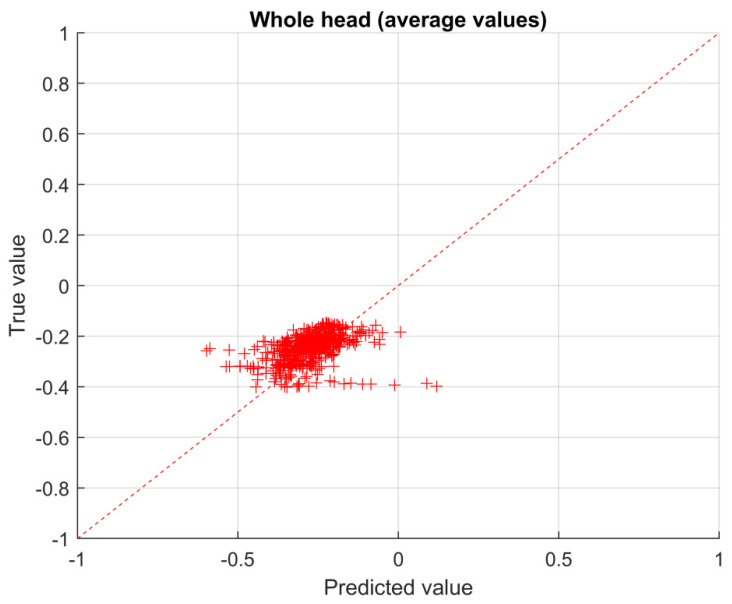
True vs. predicted values for the head region (average values).

**Figure 12:**
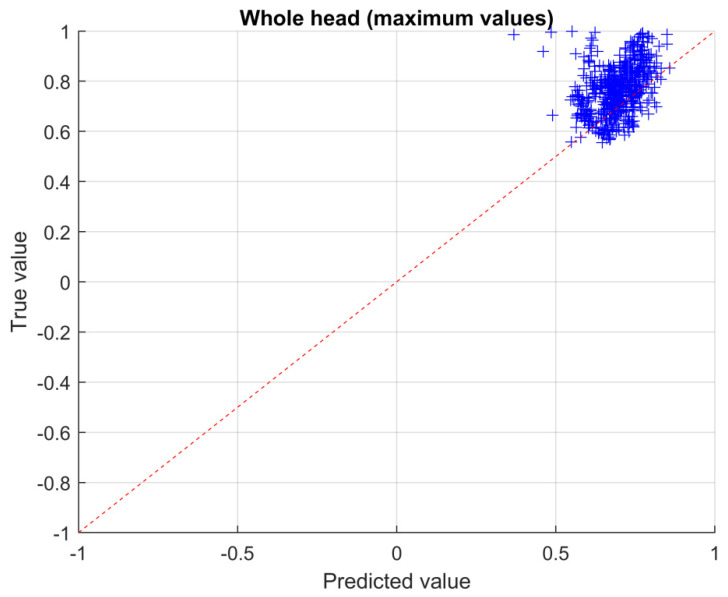
True vs. predicted values for the head region (maximum values).

The true vs predicted value plane is a graphical way to represent the accuracy of the prediction: the closer the points to the diagonal, the more accurate the solution. In particular, the normalized solutions obtained with the CNN are reported in the plane (see Figure 10, Figure 11 and Figure 12). In particular, in Figure 10 the results for the left eye are shown, while, in Figure 11 and Figure 12, the results for the head are shown, making a distinction between average values (Figure 11) and maximum values (Figure 12), respectively.

The RMSE calculated according to Equation (6), with reference to the validation set, for the left eye region (see Figure 10) is 0.12, for the whole head region, average values, is 0.057 (see Figure 11) and for the whole head region, maximum values, is 0.087 (see Figure 12).

For the sake of a comparison, the FC-NN has been trained with the same hyper-parameters and method used for the CNN training, in order to provide a comparison under the same conditions.

The RMSE calculated for the left eye and the whole head, referring to the validation set, are shown in Table 2:

The prediction given by the FC-NN is worse than the one of the CNN, and this result, in our opinion, justifies the use of a CNN, which has a more complex architecture, but it gives more accurate results.

In order to check the stability of the performance of the CNN, the CNN has been trained 30 times by varying the samples chosen for the validation set and for the training set, considering the 80/20 rule, i.e., 1110 samples for the training set and 277 for the validation set. All the runs are under the same conditions (hyper-parameters and training method). The RMSE for each run, relevant to the left eye (all values) and to the whole head (average and maximum values), is shown in Table 3, along with the MEAN and standard deviation calculated with Equations (4) and (5).

In order to show a practical use of a trained CNN for predicting the field exposure, some common and meaningful exposure angles, not-necessarily incorporated in the dataset used for the CNN training, are selected: in Table 4 a comparison between maximum and average values of power density obtained for the head region is presented; Table 5 presents data for left eye region.

It is worth to note that the results obtained by means of the FDTD model requires 4 min with 2 GB of memory, while the results obtained by means of the CNN are obtained in milliseconds.

## 4. Discussion

In the conducted numerical experiment, the average and maximum values of power densities were determined for the areas of the head and the areas of the eyeballs. The area of the head shown in Figure 5 in the form of a large ellipse contains, apart from the areas of the eyeballs, other tissues of the head. In particular, it contains areas very close to the body surface, both in front of the head and in the occipital area. This results in different average and maximum power density values for the head area and eye areas. In the case of the head area (large ellipse), the distribution of average values for different angles of the incident wave is relatively small, concentrated around −25 dB(W/m^2^). This is due to the fact that the intensity of the electric field and the energy of the incident wave do not change in the experiment. For a large sub-area of the head defined by an ellipse, the averaged value over the entire area results mainly from the amount of incident energy and tissue absorption and not from the angle of incidence of the wave. The same applies to the maximum values determined for the head area for different angles of wave incidence. Regardless of the angle of incidence of the wave, such a large area includes subareas located near the surface of the body, where the highest power density occurs. The distribution of its values results mainly from the fact that the *θ* angle was also changed in the experiment, and therefore the determined values of the power density were also affected by the tissues above and below the plane under consideration. In addition, the area of the large ellipse does not cover 100% of the area of the head and sometimes does not contain the skin layer for which, in the case of certain angles of incidence, there is an area with the maximum value of energy density.

**Figure 13:**
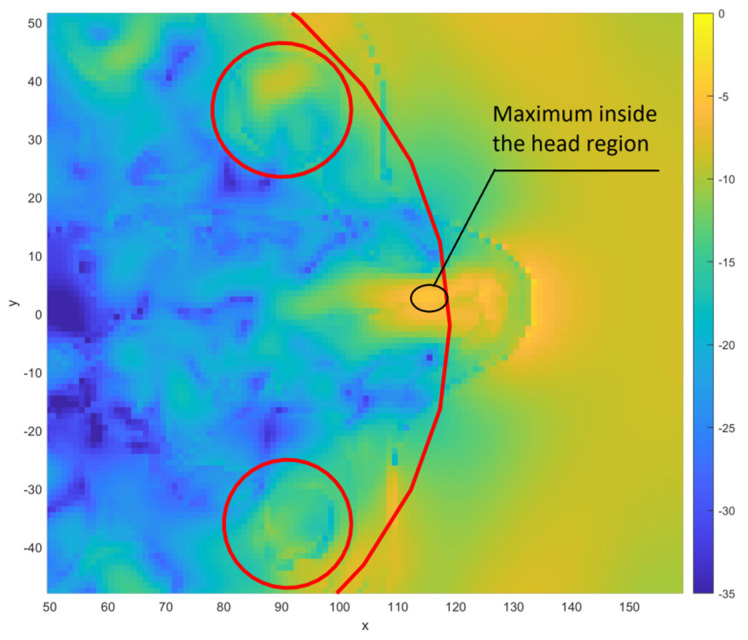
The results of power density distribution (dB) obtained for exposure to a wave coming from the direction of *φ* = 0°, *θ* = 90°. x and y dimensions given in mm. The eyes are highlighted with red circles.

In the case of parameters determined for the eye areas, there is a broader distribution of both maximum and average power densities. The eye areas are located in the front of the head, inside the head area bounded by a large ellipse. The average value of the power density determined for these areas depends to a large extent on the angle of incidence. In the case of exposure to the wave, the source of which is located in front of the head, the values (both average and maximum) calculated for the eyeball areas are higher than in the case of exposure to the wave coming from the back of the head. In the latter case, the highest energy density occurs for the areas located at the back of the head, which are not included in the calculations for the eyeballs. Therefore, in the distribution of parameter values for the eyeballs, there are values much lower than for the entire head area. The maximum values for the eyeballs are lower than the maximum values for the whole head. This is due to the fact that the head area contains places that are closer to the surface of the body. Figure 13 shows exemplary results for exposure to a wave coming from the direction of *φ* = 0°, *θ* = 90°, corresponding to the front of the head. The area with maximum power density is located near the nose. It is covered by the ellipse delineating the area of the head but not the ellipses delimiting the area of the eyeballs.

As far as the CNN performance concerns and referring to Table 2 and Table 3, it can be noted that the accuracy of the prediction of power density is acceptable: in fact, both the RMSE and the MEAN ± SD errors are rather low. Moreover, these results are rather stable over different runs. Moreover, it can be noted that these errors are calculated on the validation set, which is also used for the fine-tuning of the hyperparameters, so they are meaningful referring to the validation set, but they are not assumed to be an error indicator valid for any possible input to the trained CNN.

The results of the comparison of power density modeling using the FDTD method and the CNN are very promising. As shown in Table 4, for the head region, both average and maximum values were well approximated by the CNN. The estimation differences do not exceed a few decibels. In the case of the eye region, quite good results were also obtained. The exception is the *θ* = 45° case for which both the average and maximum values were estimated with a large error (greater than 10 dB). At a first glance, this can be due to the fact that, in the case of a wave impinging from a direction above the horizontal plane, the simulation results of the power density in the horizontal plane are substantially affected by the influence of the upper part of the head on the propagation of the EM wave. Therefore, in this case, the ‘true’ distributions of induced power are more dependent on the whole head region, while the ‘predicted’ distributions necessarily depend on the tissue distribution in the horizontal section only, the one which CNN could “learn” in the training process.

The CNN model presented in this paper was elaborated assuming that the head was exposed to a plane EM wave of a fixed frequency, amplitude, and polarization. Such an assumption is justified by the use case scenario that is radiation from 5G base stations. This, however, limits its applicability to the examination of radiation from the distant base station of assumed parameters.

## 5. Conclusions

The paper presented a preliminary result of research on the use of CNN for modeling the effect of energy absorption by the human body. A twofold remark can be put forward:

- The developed neural network makes it possible to estimate the maximum values and average values of power density for the head area and the eye area;
- The distribution of average and maximum values versus wave direction corresponds to the physical properties of tissues.

Further research is in progress on the generalization of the developed network for modeling the impact of waves of different frequencies and amplitudes.

The results of the research presented in this article are very promising. They show that CNN can be used to model very complex phenomena such as energy absorption by the human body. Despite the numerically costly process of collecting learning data, the target network allows for a significant reduction in the time needed to estimate the parameters related to the power distribution in the head and eye regions. The research methodology presented here will be used for further work, where the modeling of the absorption phenomenon at different frequencies will be investigated.

Further work will include research on the use of data obtained for cross-sections in the vertical plane in the learning process. We hope that this will improve the accuracy of the estimation for directions above the horizontal plane, which currently did not give the best results.

In our research, we took into account only one value of the wave amplitude. This is due to the fact that for the assumed combination of wave incidence angles, it was necessary to conduct many time-consuming FDTD simulations (1387). In general, the power density depends on the square of the electric field strength, but the human body is a non-linear domain whose properties strongly depend on the frequency. For such a domain, the relationship between the power density and the electric field intensity inside it is described by Cole–Cole parameters. A complex function dependent on the material frequency-dependent complex permittivity and relaxation behavior arises. As a consequence, the field inside the human body can be significantly affected by the frequency and amplitude of the applied electric field. For this reason, we plan a further investigation on how our model can handle different wave amplitudes.

## Figures and Tables

**Table 1:** Architecture of the FC-NN.

Blocks	Layers
Inputs	Image based input (size 1 × 1 × 3)
Block 1	Fully connected (size 1028)
	Batch Normalization
	Hyperbolic tangent activation function
Block 2	Fully connected (size 512)
	Batch Normalization
	Hyperbolic tangent activation function
Block 3	Fully connected (size 256)
	Batch Normalization
	Hyperbolic tangent activation function
Block 4	Fully connected (size 64)
	Batch Normalization
	Hyperbolic tangent activation function
Block 5	Fully connected (size 32)
	Batch Normalization
	Hyperbolic tangent activation function
	Fully connected (size 6)
Output	Regression layer

**Table 2:** Comparison between CNN and FC-NN in terms of RMSE.

Region	CNN	FC-NN
Left eye	0.12	0.82
Whole head (average value)	0.057	0.47
Whole head (maximum value)	0.087	0.81

**Table 3:** RMSE and MEAN ± SD for 30 trainings of the CNN.

Run	Left Eye		Whole Head			
			Average		Maximum	
	RMSE	MEAN ± SD	RMSE	MEAN ± SD	RMSE	MEAN ± SD
1	0.23	0.13 ± 0.18	0.07	0.06 ± 0.04	0.11	0.09 ± 0.06
2	0.11	0.09 ± 0.07	0.34	0.21 ± 0.27	0.09	0.08 ± 0.05
3	0.19	0.14 ± 0.13	0.10	0.08 ± 0.06	0.10	0.09 ± 0.06
4	0.12	0.10 ± 0.07	0.32	0.18 ± 0.27	0.12	0.09 ± 0.07
5	0.10	0.08 ± 0.06	0.37	0.22 ± 0.29	0.15	0.09 ± 0.12
6	0.11	0.09 ± 0.07	0.07	0.05 ± 0.04	0.12	0.09 ± 0.08
7	0.10	0.07 ± 0.06	0.06	0.04 ± 0.04	0.12	0.09 ± 0.08
8	0.11	0.09 ± 0.07	0.08	0.06 ± 0.05	0.10	0.08 ± 0.06
9	0.22	0.12 ± 0.18	0.06	0.05 ± 0.04	0.12	0.09 ± 0.07
10	0.14	0.10 ± 0.10	0.08	0.06 ± 0.05	0.11	0.09 ± 0.06
11	0.21	0.13 ± 0.17	0.06	0.05 ± 0.03	0.11	0.09 ± 0.06
12	0.12	0.09 ± 0.08	0.42	0.28 ± 0.32	0.08	0.07 ± 0.05
13	0.12	0.09 ± 0.08	0.36	0.23 ± 0.28	0.12	0.09 ± 0.08
14	0.14	0.11 ± 0.09	0.32	0.19 ± 0.27	0.13	0.09 ± 0.09
15	0.10	0.08 ± 0.06	0.04	0.03 ± 0.03	0.10	0.09 ± 0.06
16	0.14	0.11 ± 0.09	0.09	0.07 ± 0.05	0.15	0.13 ± 0.07
17	0.18	0.12 ± 0.13	0.06	0.05 ± 0.04	0.15	0.11 ± 0.10
18	0.09	0.07 ± 0.06	0.06	0.05 ± 0.04	0.14	0.09 ± 0.11
19	0.10	0.08 ± 0.06	0.06	0.05 ± 0.04	0.11	0.08 ± 0.08
20	0.12	0.10 ± 0.07	0.07	0.06 ± 0.04	0.11	0.08 ± 0.07
21	0.25	0.14 ± 0.20	0.07	0.05 ± 0.05	0.10	0.08 ± 0.06
22	0.14	0.10 ± 0.10	0.07	0.05 ± 0.04	0.13	0.10 ± 0.09
23	0.12	0.09 ± 0.07	0.06	0.05 ± 0.04	0.19	0.12 ± 0.15
24	0.28	0.15 ± 0.23	0.06	0.05 ± 0.04	0.10	0.08 ± 0.05
25	0.12	0.09 ± 0.08	0.11	0.08 ± 0.07	0.15	0.11 ± 0.10
26	0.14	0.10 ± 0.10	0.06	0.05 ± 0.04	0.12	0.09 ± 0.07
27	0.28	0.16 ± 0.22	0.08	0.05 ± 0.06	0.09	0.08 ± 0.06
28	0.11	0.09 ± 0.07	0.06	0.04 ± 0.04	0.10	0.08 ± 0.06
29	0.11	0.09 ± 0.06	0.07	0.06 ± 0.03	0.09	0.08 ± 0.05
30	0.25	0.15 ± 0.20	0.07	0.06 ± 0.04	0.11	0.09 ± 0.07

**Table 4:** Comparison between maximum and average values of power density obtained for the head region.

*φ* [°]	*θ* [°]	CNN		FDTD	
		Max [dB]	Average [dB]	Max [dB]	Average [dB]
0	90	−6.60	−23.43	−4.80	−23.67
0	45	−5.42	−25.63	−7.11	−25.49
90	90	−9.40	−24.38	−8.31	−23.20
90	45	−4.90	−26.44	−8.45	−23.46
180	90	−9.71	−26.18	−10.07	−26.01
180	45	−7.71	−28.84	−7.93	−24.61

**Table 5:** Comparison between maximum and average values of power density obtained for the left eye region.

*φ* [°]	*θ* [°]	CNN		FDTD	
		Max [dB]	Average [dB]	Max [dB]	Average [dB]
0	90	−8.70	−14.30	−8.04	−14.83
0	45	−10.70	−12.67	−21.80	−27.35
90	90	−12.99	−18.66	−11.62	−18.09
90	45	−22.19	−25.28	−22.44	−27.47
180	90	−22.53	−32.44	−23.81	−31.32
180	45	−23.10	−33.76	−9.68	−16.75

## Data Availability

The simulation results are available upon email request.
